# Efficacy of Recombinant Human Endostatin plus Neoadjuvant Chemotherapy for Osteosarcoma and Its Influence on Serum VEGF and MMP-9 Levels

**DOI:** 10.1155/2023/8161683

**Published:** 2023-02-25

**Authors:** Wei Sui, Guanghui Lin

**Affiliations:** Department of Orthopaedics, North District, Xiangyang Central Hospital Affiliated to Hubei University of Arts and Sciences, Xiangyang 441002, Hubei, China

## Abstract

**Objective:**

To investigate the efficacy of recombinant human endostatin (rh-Endo) plus neoadjuvant chemotherapy (NACT) for osteosarcoma (OSA) and its influence on serum vascular endothelial growth factor (VEGF) and matrix metalloproteinase-9 (MMP-9).

**Methods:**

The case data of 141 OSA patients presented to the North District, Xiangyang Central Hospital Affiliated to Hubei University of Arts and Sciences from January 2018 to June 2019, were analyzed retrospectively. Patients receiving NACT (methotrexate + ifosfamide + adriamycin) were assigned into the control group (CNG; *n* = 65), while those treated with rh-Endo plus NACT were included in the combination group (CMG; *n* = 76). The following aspects were compared: clinical efficacy, serum tumor markers, serum VEGF and MMP-9 contents, inflammatory factors, incidence of adverse reactions, limb function scores at 6 months of follow-up, and prognostic quality of life (QOL).

**Results:**

A statistically higher overall response rate (ORR) was determined in CMG versus CNG (84.2% vs. 64.6%, *P* < 0.05). The pretreatment serum bone alkaline phosphatase (BALP), insulin-like growth factor (IGF)-1, serum amyloid A (SAA), VEGF, MMP-9, C-reactive protein (CRP), tumor necrosis factor (TNF)-*α*, and interleukin (IL)-10 levels differed insignificantly between the two cohorts (*P* > 0.05); while except IL-10 that showed increased expression in both cohorts and was comparatively higher in CMG, the other 8 parameters reduced in both cohorts after 2 weeks of drug withdrawal, and the reduction of each parameter was more significant in CMG (*P* < 0.05). The total adverse reaction rate was 30.2% in CMG, which was higher than that of 36.9% in CNG, albeit without a statistical difference (*P* > 0.05). An evidently higher 2-year survival rate was determined in CMG (*P* < 0.05).

**Conclusions:**

rh-Endo plus NACT is more effective than NACT alone in the treatment of osteosarcoma, which can validly restore the balance of vascular endothelial cells, reduce inflammation, and is worth promoting in clinic.

## 1. Introduction

Osteosarcoma (OSA) is the most commonly seen primary bone malignancy derived from primitive osteoid mesenchymal cells, and its pathogenesis is closely related to puberty hormone changes and bone growth [1, 2]. The onset age of OSA presents a bimodal distribution, most of which occurs in adolescents aged 10–14 and the elderly aged over 60 [3]. In adolescence, the disease often occurs in the long bone metaphysis of the extremities, such as the distal femur and the proximal tibia [4, 5]. For the elderly, however, the predilection sites are diverse, which can also occur in the craniofacial region [6]. OSA has a high propensity to metastasize, specifically to the lungs, with more than 85% of patients developing lung metastasis at the time of initial treatment [7]. At present, surgery combined with adjuvant chemotherapy remains the mainstay treatment option for OSA. Although this strategy has improved OSA patients' 5-year survival to some extent, the survival is still extremely low, especially for those with metastatic and recurrent OSA, with a survival rate less than 20% [8, 9]. In addition to distant metastasis, the prime reasons for an adverse prognosis are poor response to chemotherapy and drug resistance to chemotherapeutics [10]. Therefore, there is a growing demand for new drugs and treatments for OSA.

Precise surgical resection and combined neoadjuvant chemotherapy (NACT) established by methotrexate (MTX), adriamycin (ADM), cisplatin, and ifosfamide (IFO) can improve OSA patients' 5-year survival to 60–70% [11]. Today, most patients receive preoperative NACT and postoperative adjuvant chemotherapy. Unfortunately, the utility of many chemotherapeutics is limited by their toxicity and side effects. For example, MTX can be fatal in excess of a certain dose [12], ADM-induced cardiomyopathy can cause permanent irreversible damage to the heart of juvenile patients [13], and the addition of ifosfamide (IFO) can increase the toxicity of chemotherapy [14].

In recent years, some new treatments for OSA have emerged, with a growing number of drugs developed focusing on the pathogenesis of OSA. Previous literature has revealed that OSA, like other tumors, is dependent on angiogenesis [15]. Therefore, antiangiogenesis has become a new therapeutic approach. Recombinant human endostatin (rh-Endo) is a molecular targeted therapeutic drug, which has the effect of inhibiting tumor growth, invasion, and metastasis with little damage to normal vascular endothelial cells; moreover, it can specifically inhibit tumor tissues, with less toxic and side effects and strong pertinence [16]. Antiangiogenic therapeutic strategies are divided into two categories: one is the therapeutic strategy that directly produces cytotoxicity on endothelial cells, and the other is the therapeutic strategy that indirectly acts by eliminating key proangiogenic factors such as vascular endothelial growth factor (VEGF) [17]. As an essential target of matrix metalloproteinases (MMPs) and other angiogenesis factors and proteases, VEGF is critical in tumor metastasis [18]. Among MMPs, MMP-9 is the product of VEGF receptor (VEGF-R) activation, and the expression of this downstream factor shows the potential to be a surrogate marker of VEGF-R activity [19]. Studies have shown that the antiangiogenesis of rh-Endo can prevent metastatic OSA from further progressing [20]. Yet there is little information directly correlating serum MMP-9 levels with VEGF activity in OSA. Accordingly, this study's novelty and aim lie in the investigation of the therapeutic effect of rh-Endo plus NACT for OSA and its influence on serum VEGF and MMP-9 levels.

## 2. Materials and Methods

### 2.1. Research Participants

**Figure 1:**
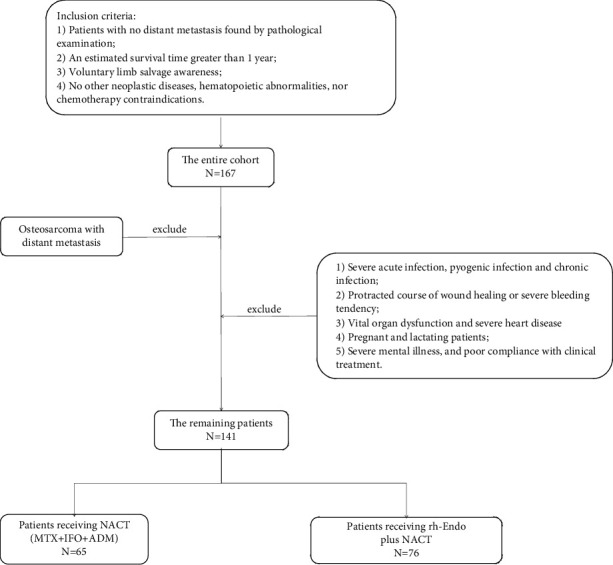
Flowchart for screening patients.

The clinical records of 141 patients with a confirmed diagnosis of OSA by symptoms, signs, imaging, and pathology and treated in the North District at Xiangyang Central Hospital Affiliated to Hubei University of Arts and Sciences from January 2018 to June 2019 were analyzed retrospectively (Figure 1). Patients receiving NACT (MTX + IFO + ADM) were assigned into control group (CNG; *n* = 65), while those treated with rh-Endo plus NACT were included in the combination group (CMG; *n* = 76). Inclusion criteria were included as follows: Patients with no distant metastasis found by pathological examination, an estimated survival time greater than 1 year, voluntary limb salvage awareness, and no other neoplastic diseases, hematopoietic abnormalities, or chemotherapy contraindications. The exclusion criteria included OSA with distant metastasis, severe acute infection, pyogenic infection, and chronic infection, protracted course of wound healing or severe bleeding tendency, vital organ dysfunction, severe heart disease, pregnant and lactating patients, severe mental illness, and poor compliance with clinical treatment. The two cohorts had similar general clinical data, indicating comparability (Table 1). This Ethics Committee of *Xiangyang Central Hospital Affiliated to Hubei University of Arts and Sciences* has given its approval for conducting this study.

### 2.2. Treatment Regimens

Both groups received the MMIT regime for NACT: MTX (Haizheng Pfizer Pharmaceutical Co., Ltd., 5 mg × 5 injections) was injected intravenously at 10 g/m^2^ in the first and second weeks; in the third week, IFO (Guangdong Lingnan Pharmaceutical Co., Ltd., 1 g for injection) was given by intravenous drip at 2 g/m^2^ for 5 days, and in the third week, intravenous ADM (30 mg/m^2^ Shanxi Pude Pharmaceutical Co., Ltd., 10 mg × 10 injections) was administered for 3 days. With 4 weeks as a cycle, the chemotherapy was performed for two cycles. On this basis, patients in CMG were treated with rh-Endo (Shangdong Simcere Pharmaceutical Group Ltd., 15 mg: 3 mL), 15 mg per time intravenously, for 2 weeks. The granisetron, dexamethasone, and metoclopramide were routinely used for antiemetic treatment during chemotherapy, and a subcutaneous injection of colony-stimulating factor was used prophylactically after chemotherapy to protect the bone marrow. Both groups were treated for 2 cycles, and surgery was performed 2 weeks after drug withdrawal.

### 2.3. Outcome Measures

#### 2.3.1. Clinical Efficacy Assessment

The therapeutic effect at 12 weeks was evaluated by referring to *the WHO Response Evaluation Criteria in Solid Tumors* [21]. Complete resolution of the lesion was considered a complete response (CR); ≥50% decrease of the maximum bi-diameter product of the lesion that lasted for more than 4 weeks was deemed a partial response (PR); stable disease (SD) is indicated if the maximum bi-diameter product of the lesion was reduced by < 50% with no new lesions for over 4 weeks;: ≥25% increase of the maximum bi-diameter product of the lesion or occurrence of new lesions was deemed progressive disease (PD). The effective rate = (CR + PR) cases/total cases × 100%.

#### 2.3.2. Detection of OSA-Related Tumor Markers

From all patients, fasting peripheral venous blood was collected before treatment and two weeks after drug withdrawal, for anticoagulation treatment and serum separation. Serum levels of OSA-associated tumor markers (bone alkaline phosphatase (BALP), insulin-like growth factor (IGF)-1, and serum amyloid A (SAA)) were detected by enzyme-linked immunosorbent assay (ELISA) kits offered by Beijing Ruida Henghui Science and Technology Development Co., Ltd.

#### 2.3.3. Serum VEGF and MMP9 Determination

After collecting from patients before treatment and two weeks after drug withdrawal, fasting peripheral venous blood was treated with anticoagulation treatment and serum separation, to measure serum VEGF and MMP-9 concentrations using VEGF ELISA kit offered by Beijing Ruida Henghui Science and Technology Development Co., Ltd. and MMP-9 ELISA kits by Wuhan Boster Biological Technology Co., Ltd.

#### 2.3.4. Serum Inflammatory Cytokines

Fastening peripheral venous blood of all patients was collected before treatment and two weeks after drug withdrawal, respectively, for anticoagulation treatment and serum separation. Serum C-reactive protein (CRP), tumor necrosis factor (TNF)-*α*, and interleukin (IL)-10 were detected using ELISA with kits all provided by Wuhan Boster Biological Technology Co., Ltd.

#### 2.3.5. Evaluation of Limb Function

Patients' limb activity function, assessed by the Musculoskeletal Tumor Society 93 (MSTS 93) scoring system [22] from six aspects (pain, level of activity and restriction, emotional acceptance, use of orthopedic supports, walking ability, and gait), was compared at 6 and 12 months of follow-up, respectively.

#### 2.3.6. Quality of Life (QOL) Scores

Patients' QOL was assessed before treatment and at one-yearfollow-up using the European Organization for Research and Treatment of Cancer Quality of Life Questionnaire (EORTC QLQ-C30) [23] from five domains, namely, physical, cognitive, role, social, and emotional functioning. A higher score indicates a better QOL.

#### 2.3.7. Adverse Reactions

Adverse reactions that occurred during treatment, including gastrointestinal reactions, leucopenia, thrombocytopenia, peripheral neurotoxicity, liver function injury, renal function damage, bone marrow suppression, and fever, were recorded.

#### 2.3.8. Survival Outcome

A two-yearfollow-up was performed on all patients, and the overall survival (time from treatment initiation to the completion of follow-up or patient death) was recorded.

### 2.4. Statistical Methods

The sample size was calculated using PASS 15.0, which was based on the assumption that the 2-year survival probability in the CNG was 53%. The target sample size was required to be at least 98 (alpha error 0.05; power 0.8) in both groups (49 for each), with a dropout rate of 20%. It was calculated that at least 124 cases (62 cases in each group) were needed for the study.

SPSS 22.0 (SPSS Inc., Chicago, IL, USA) processed the collected data, and GraphPad Prism 8.0 (GraphPad Software, San Diego, CA, USA) visualized it. The Kolmogorov-Smirnov Z normal distribution test and Levene variance homogeneity test shall be passed before the test. For intragroup before-after comparisons of continuous variables represented by mean ± standard deviation, the paired sample *t*-test was used; for between-group comparisons, the independent sample *t*-test was adopted; for multigroup comparisons, a one-way ANOVA was used followed by the Bonferroni post-hoc test (*α* = 0.05). Categorical variables, described as *n* (%), were analyzed using the chi-square test. The significance level was *P* < 0.05.

## 3. Results

### 3.1. Clinical Efficacy

The two cohorts differed significantly in the clinical efficacy, with a higher overall response rate (ORR) in CMG than in CNG (84.2% vs. 64.6%, *P* < 0.05).  Table 2.

### 3.2. Levels of Tumor Markers

**Figure 2:**
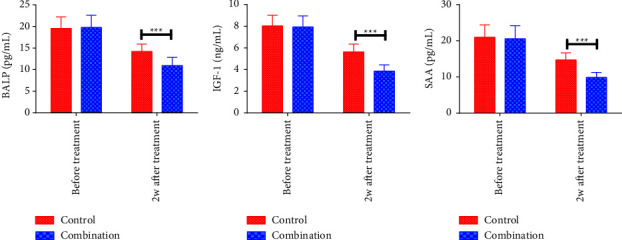
Comparison of tumor marker levels. (a) Serum BALP levels; (b) serum IGF-1 levels; (c) serum SAA levels; ^*∗∗∗*^*P* < 0.001.

The pretreatment serum BALP, IGF-1, and SAA levels differed insignificantly between groups (*P* > 0.05). Two weeks after treatment discontinuation, serum BALP, IGF-1, and SAA were reduced in both cohorts, with more marked reductions in CMG compared with CNG (*P* < 0.05).  Figure 2.

### 3.3. Serum VEGF and MMP-9 Levels

**Figure 3:**
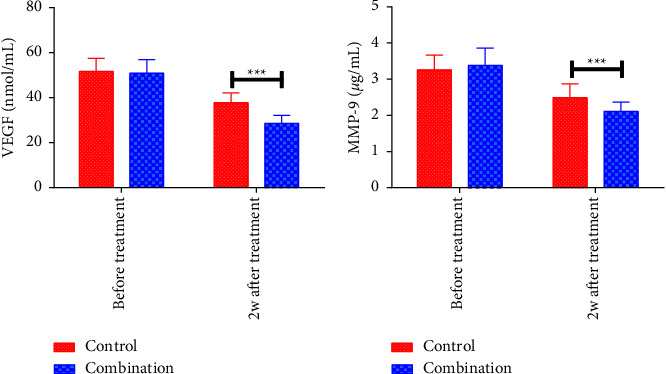
Comparison of serum VEGF and MMP-9 levels. (a) Serum VEGF levels; (b) serum MMP-9 levels; ^*∗∗∗*^*P* < 0.001.

The pretreatment serum VEGF and MMP-9 levels differed insignificantly between the two cohorts (*P* > 0.05). Two weeks after treatment discontinuation, the two parameters decreased in both cohorts, and the decreases were more obvious in CMG (*P* < 0.05).  Figure 3.

### 3.4. Serum Inflammatory Factors

**Figure 4:**
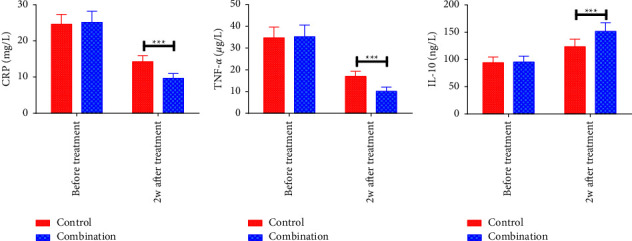
Comparison of serum inflammatory factors. (a) Serum CRP levels; (b) serum TNF-*α* levels; (c) serum IL-10 levels; ^*∗∗∗*^*P* < 0.001.

No significant intergroup differences were observed regarding the pretreatment serum CRP, TNF-*α*, and IL-10 levels (*P* > 0.05). Two weeks after treatment discontinuation, CRP and TNF-*α* levels decreased in both cohorts while IL-10 increased, and the changes were more obvious in CMG (*P* < 0.05).  Figure 4.

### 3.5. Adverse Reactions

The adverse reactions found in CMG were gastrointestinal reactions (4 cases), leukopenia (2 cases), thrombocytopenia (4 cases), peripheral neurotoxicity (2 cases), liver function injury (3 cases), renal function injury (2 cases), myelosuppression (2 cases), and fever (4 cases), with an overall incidence rate of 30.2%, while the corresponding data in CNG were 5, 3, 4, 2, 2, 2, 3, and 3 cases, respectively, with an overall incidence of 36.9%. The two groups differed insignificantly in the incidence of each of the above adverse reactions (*P* > 0.05) (Table 3).

### 3.6. Limb Function

After 6 months of follow-up, CMG showed a statistically higher MSTS 93 score than CNG with an obviously higher excellent rate (79.0% vs. 63.1%, *P* < 0.05).  Table 4.

### 3.7. QOL

Patients' QOL was compared based on scores of physical functioning, cognitive functioning, role functioning, social functioning, and emotional functioning after one year of follow-up. The results revealed no marked difference in pretreatment QOL between groups (*P* > 0.05). After one year of follow-up, the scores of the various dimensions in CMG were obviously higher, suggesting a better QOL in patients receiving rh-Endo plus NACT (*P* < 0.05).  Table 5.

### 3.8. Overall Survival Rate

**Figure 5:**
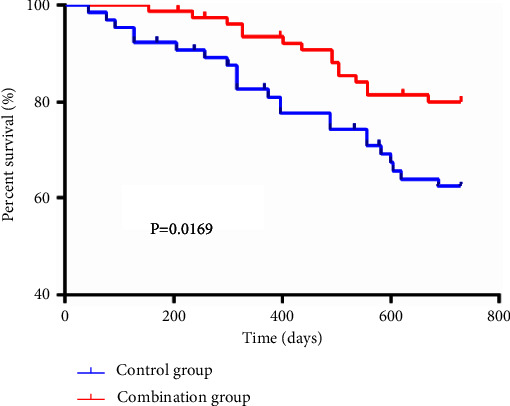
Overall survival rate of two groups.

All patients underwent a 2-year postoperative follow-up. In CMG, 15 patients died with 5 deleted cases. In CNG, 23 patients died with 7 deleted cases. The 2-year survival rate was statistically higher in CMG versus CNG (*P* = 0.0169).  Figure 5.

## 4. Discussion

The specific etiology of OSA has not been clarified. But relevant research reports have revealed its correlation with immune system disorders, radiation, virus infections, and genetic factors [24, 25]. After the episode, there will be intermittent pain in the affected part, which will be aggravated with the progression of the disease. Finally, painkillers will not help, and the tumor may develop and metastasize to the lungs to harm the patient's respiratory function, leading to high disability and mortality [26]. Chemotherapy is currently one of the most effective methods in addition to surgical treatment, but chemotherapeutics generally lack targeting and have extensive cytotoxicity. When chemotherapeutics enter the body, normal cells, especially those with vigorous metabolism, can be damaged to varying degrees. MMIT is a commonly used NACT regimen for OSA in the clinic to kill tumor cells, in which MTX can inhibit DNA synthesis mainly by mediating the S phase of the cell cycle, IFO can cross-link with DNA to exert cytotoxicity, and ADM can inhibit the synthesis of RNA and DNA [27, 28]. rh-Endo injection is a therapeutic drug with molecular targeting function, which can specifically block the formation of tumor blood vessels, thus inhibiting tumor cell growth, infiltration and metastasis. Previous studies have shown that [29], the combination of rh-Endo in NACT can significantly prolong progression-free time, inhibit disease progression, and reduce recurrence.

The present research aims to explore the efficacy of rh-Endo plus NACT for OSA and its influence on serum VEGF and MMP-9 levels. The results revealed a statistically higher ORR in CMG than in CNG (84.2% vs. 64.6%). Moreover, serum tumor markers BALP, IGF-1, and SAA were reduced in both cohorts after two weeks of chemotherapy drug withdrawal, with lower levels in CMG. The detection of tumor markers is a routine means to reflect the tumor load of malignant lesions. BALP is a marker for OSA cell growth, which is upregulated in more than 40% of OSA patients [30]. IGF-1 participates in OSA cell division and multiplication, and its high expression can promote the distant metastasis of OSA cells [31]. As to SAA, it is one of the markers that distinguish benign from malignant OSA, and is upregulated in OSA tissues and released into the blood [32]. After NACT plus rh-Endo intervention, these indexes were significantly reduced, with a reduction degree better than that of NACT alone. In addition, serum VEGF and MMP-9 were notably lower in CMG than in CNG two weeks after stopping chemotherapy. VEGF is a key factor in tumor angiogenesis, which mainly stimulates the proliferation and differentiation of vascular endothelial cells by autocrine or paracrine, and participates in every link of tumor cells in the form of abnormally high expression in many organ tumors of the human body [33]. MMP-9 can increase tumor cell invasion and metastasis and promote vascular endothelial cell migration to form new blood vessels through the degradation of the extracellular matrix and basement membrane [34]. The results of this study, together with many previous studies, prove that VEGF and MMP-9 are highly expressed in OSA patients, and rh-Endo can effectively reduce their expression. Furthermore, we found that two weeks after drug withdrawal, serum CRP and TNF-*α* levels decreased and IL-10 levels increased, and the relief of inflammation was more significant in CMG. CRP and TNF-*α* are both vital pro-inflammatory factors in the human body, and their elevated expression is not only related to OSA-induced inflammatory responses but also to the invasive proliferation, infiltration, and injury induced by tumor cells [35, 36], while IL-10 is an essential anti-inflammatory factor. Under normal conditions, the levels of anti-inflammatory and pro-inflammatory factors will form a dynamic balance [37]. However, IL-10 will be inhibited when there is a malignancy, and will be gradually increased after treatment. By comparing the incidence of adverse reactions produced by the two treatment regimens, we found no significant difference between the two cohorts. Moreover, after 6 months and one year of follow-up, patients' limb function and QOL were better in CMG, suggesting that rh-Endo can improve the limb function and QOL of patients. However, this research still shows some shortcomings. First, due to the nature of being a single-center retrospective analysis, a multicenter prospective clinical trial is needed for confirmation. Second, a randomized double-blind clinical trial is required to validate the findings as the chemotherapy regimen in this study is not randomly selected. Third, the sample size is relatively small, so it is necessary to further assess the effectiveness and safety of rh-Endo plus NACT in OSA. We will increase patient samples covering different stages or pathological types, and extend the follow-up time to further evaluate the effectiveness of this combination therapy for OSA.

## 5. Conclusion

To sum up, rh-Endo plus NACT is effective for OSA, which can help restore the balance of inflammatory factor expression in patients, inhibit tumor angiogenesis to reduce tumour invasion and metastasis, and improve limb function and QOL of patients, providing a feasible treatment plan for follow-up treatment.

## Figures and Tables

**Table 1:** Baseline data.

	Control group (*n* = 65)	Combination group (*n* = 76)	*χ * ^2^/*t*	*P*
Gender (*n* (%))			0.0499	0.8231
Male	39 (60.0)	47 (61.8)		
Female	26 (40.0)	29 (38.2)		
Average age (years)	23.74 ± 2.48	24.05 ± 2.64	0.7146	0.4760
BMI (kg/m^2^)	20.97 ± 1.79	21.16 ± 1.94	0.6006	0.5491
Tumor location			0.8834	0.8294
Ulna	12 (18.5)	16 (21.1)		
Perone	17 (26.2)	15 (19.7)		
Tibia	20 (30.7)	26 (34.2)		
Femur	16 (24.6)	19 (25.0)		
Pathological type			1.3992	0.4970
Osteoblast	41 (63.1)	55 (72.4)		
Chondroblast	14 (21.5)	12 (15.8)		
Fibroblast	10 (15.4)	9 (11.8)		
Enneking stage			0.7495	0.6875
IIA	10 (15.4)	8 (10.5)		
IIB	48 (73.8)	59 (77.6)		
III	7 (10.8)	9 (11.9)		

**Table 2:** Clinical efficacy.

	CR	PR	SD	PD	Overall response rate
Control group (*n* = 65)	20 (30.8)	22 (33.8)	13 (20.0)	10 (15.4)	42 (64.6)
Combination group (*n* = 76)	38 (50.0)	26 (34.2)	8 (10.5)	4 (5.3)	64 (84.2)
*χ * ^2^	8.8773				7.2091
*P*	0.0310				0.0073

**Table 3:** Comparison of adverse reactions.

	Gastrointestinal reaction	Leukopenia	Thrombocytopenia	Peripheral neurotoxicity	Liver function injury	Renal function injury	Myelosuppression	Fever	Overall incidence
Control group (*n* = 65)	5 (7.6)	3 (4.6)	4 (6.2)	2 (3.1)	2 (3.1)	2 (3.1)	3 (4.6)	3 (4.6)	24 (36.9)
Combination group (*n* = 76)	4 (5.3)	2 (2.6)	4 (5.3)	2 (2.6)	3 (3.9)	2 (2.6)	2 (2.6)	4 (5.3)	23 (30.2)
*χ * ^2^	0.3460	0.4031	0.0519	0.0252	0.0776	0.0252	0.4031	0.0312	0.6993
*P*	0.5564	0.5255	0.8197	0.8738	0.7806	0.8738	0.5255	0.8599	0.4030

**Table 4:** Comparison of limb function.

	Score	Excellent	Good	Fair	Poor	Excellent rate
Control group (*n* = 65)	24.12 ± 2.31	29 (44.6)	12 (18.5)	14 (21.5)	10 (15.4)	41 (63.1)
Combination group (*n* = 76)	27.64 ± 2.84	43 (56.6)	17 (22.4)	7 (9.2)	9 (11.8)	60 (79.0)
*χ * ^2^/*t*	7.9847					4.3431
*P*	<0.0001					0.0372

**Table 5:** Comparison of quality of life.

	Physical functioning		Cognitive function		Role functioning		Social functioning		Emotional functioning	
	Before treatment	One year after treatment	Before treatment	One year after treatment	Before treatment	One year after treatment	Before treatment	One year after treatment	Before treatment	One year after treatment
Control group (*n* = 65)	64.26 ± 5.97	77.69 ± 7.51	62.54 ± 5.53	76.84 ± 6.54	61.25 ± 5.23	78.64 ± 6.02	63.48 ± 6.08	80.14 ± 6.87	67.58 ± 6.31	81.54 ± 5.69
Combination group (*n* = 76)	65.04 ± 6.28	85.36 ± 7.33	62.78 ± 5.39	84.26 ± 6.45	61.87 ± 5.34	86.48 ± 6.69	64.03 ± 6.22	87.64 ± 7.18	68.04 ± 6.72	87.15 ± 6.13
*t*	0.7520	6.1239	0.2604	6.7656	0.6938	7.2619	0.5288	6.3067	0.4167	5.5983
*P*	0.4533	<0.0001	0.7949	<0.0001	0.4889	<0.0001	0.5977	<0.0001	0.6775	<0.0001

## Data Availability

The labeled dataset used to support the findings of this study are available from the corresponding author upon request.
